# University of Pennsylvania

**Published:** 1851-10

**Authors:** 


### UNIVERSITY OF PENNSYLVANIA.

In the report of the Faculty of the University of Pennsylvania for the past session, perfect satisfaction is expressed with the success of the experiment of a lengthened term. "Certainly," they say, "if a measure can be judged by its fruits, this must, so far as experience has yet determined, be considered as highly efficacious."

We are gratified to see this declaration. We have from the first felt favorably disposed to the extension of the lecture-term, and since the innovation has proved successful in the parent school of the country, we hope to see it gradually introduced into the others. Y.

